# Uridine Affects Liver Protein Glycosylation, Insulin Signaling, and Heme Biosynthesis

**DOI:** 10.1371/journal.pone.0099728

**Published:** 2014-06-11

**Authors:** Yasuyo Urasaki, Giuseppe Pizzorno, Thuc T. Le

**Affiliations:** 1 Nevada Cancer Institute, Las Vegas, Nevada, United States of America; 2 Desert Research Institute, Las Vegas, Nevada, United States of America; Institute for Nutritional Sciences, China

## Abstract

Purines and pyrimidines are complementary bases of the genetic code. The roles of purines and their derivatives in cellular signal transduction and energy metabolism are well-known. In contrast, the roles of pyrimidines and their derivatives in cellular function remain poorly understood. In this study, the roles of uridine, a pyrimidine nucleoside, in liver metabolism are examined in mice. We report that short-term uridine administration in C57BL/6J mice increases liver protein glycosylation profiles, reduces phosphorylation level of insulin signaling proteins, and activates the HRI-eIF-2α-ATF4 heme-deficiency stress response pathway. Short-term uridine administration is also associated with reduced liver hemin level and reduced ability for insulin-stimulated blood glucose removal during an insulin tolerance test. Some of the short-term effects of exogenous uridine in C57BL/6J mice are conserved in transgenic *UPase1* ^−/−^ mice with long-term elevation of endogenous uridine level. *UPase1* ^−/−^ mice exhibit activation of the liver HRI-eIF-2α-ATF4 heme-deficiency stress response pathway. *UPase1* ^−/−^ mice also exhibit impaired ability for insulin-stimulated blood glucose removal. However, other short-term effects of exogenous uridine in C57BL/6J mice are not conserved in *UPase1* ^−/−^ mice. *UPase1* ^−/−^ mice exhibit normal phosphorylation level of liver insulin signaling proteins and increased liver hemin concentration compared to untreated control C57BL/6J mice. Contrasting short-term and long-term consequences of uridine on liver metabolism suggest that uridine exerts transient effects and elicits adaptive responses. Taken together, our data support potential roles of pyrimidines and their derivatives in the regulation of liver metabolism.

## Introduction

Uridine is a pyrimidine nucleoside that has the ability to affect liver energy metabolism. Uridine is produced via several reversible reactions including de-phosphorylation of a uridine monophosphate, de-amination of a cytidine, or combination of a uracil and a ribose-1-phosphate [1]. Uridine homeostasis is regulated in part by uridine phosphorylase, an enzyme that catalyzes the reversible conversion of uridine to uracil [2]. *UPase1*-TG mice with ubiquitous genetic knock-in of a gene encoding for UPase1 exhibit depleted plasma and liver uridine concentration [3]. *UPase1*-TG mice also exhibit intrinsic liver lipid accumulation [3], [4]. In contrast, *UPase1* ^−/−^ mice with ubiquitous genetic knock-out of a gene encoding for UPase1 exhibit elevated plasma and liver uridine concentration [5], [6]. *UPase1* ^−/−^ mice are protected against fatty liver condition induced by a number of drugs with different acting mechanisms [7]–[9]. Dietary uridine supplementation is able to suppress intrinsic fatty liver in *UPase1*-TG mice or drug-induced fatty liver in C57BL/6J mice [3], [7], [9]. Clearly, uridine exerts protective effects against liver lipid accumulation; however, the underlying mechanisms have not been delineated.

Uridine is a versatile molecule that exerts multi-targeted effects because it can be used to produce other biologically active molecules. Via the pyrimidine salvage pathway, uridine can be salvaged into uridine triphosphate (UTP) and cytidine triphosphate (CTP) [1]. Combination of UTP with glucose-1-phosphate produces uridine diphosphate glucose (UDPG), which is a basic building block for glycogen biosynthesis [10]. Combination of UTP with N-acetylglucosamine (GlcNAc) produces UDP-GlcNAc, which is a donor substrate for protein glycosylation [11]. Combination of CTP with phosphocholine produces cytidine diphosphocholine (CDPC), which is an essential molecule for membrane phospholipid biosynthesis [12], [13]. On the other hand, uridine catabolism produces β-alanine and acetyl-CoA [1]. Acetyl-CoA is an important molecule in cellular energy metabolism and in the biosynthesis of the neurotransmitter acetylcholine [14], [15]. Acetyl-CoA is also a donor substrate for protein lysine acetylation, a mode of nutrient-sensitive protein post-translational modification [16], [17]. Therefore, uridine has the ability to affect a wide range of biological processes.

In recent years, clinical data from several independent labs revealed a positive correlation between plasma uridine concentration and insulin resistance in humans [18], [19]. This correlation has also been reported in rodents [11]. However, the mechanistic link between uridine and insulin signaling activity has not been elucidated. In this study, we screen for the effects of uridine on liver metabolism with specific focuses on glucose utilization and insulin signaling activity. C57BL/6J mice are fed with uridine supplemented diet for 5 days to evaluate short-term effects of uridine. Long-term effects of uridine are evaluated in transgenic *UPase1* ^−/−^ and *UPase1*-TG mice with disrupted uridine homeostasis.

## Results

**Figure 1:**
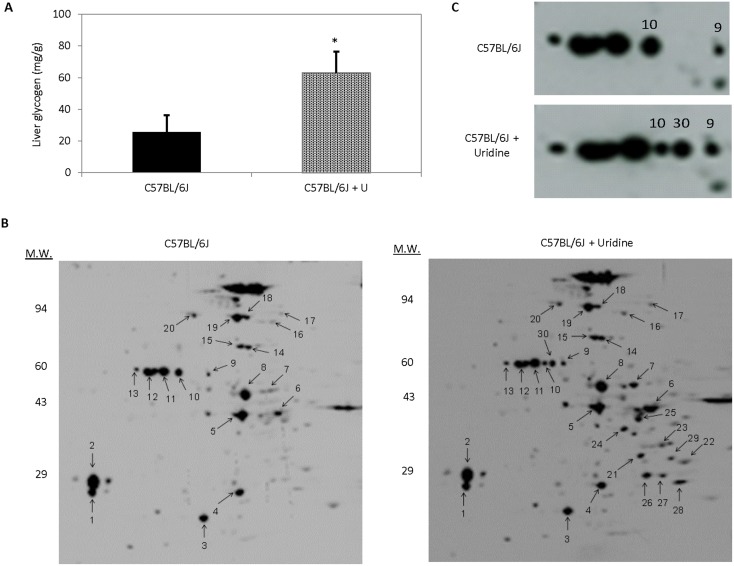
Uridine supplementation increases liver glycogen content and protein O-linked glycosylation profiles. (**A**) Liver glycogen as a function of dietary uridine supplementation. Error bars are standard deviations across 9 liver samples evaluated. Asterisk indicates P<0.05. (**B**) 2D Western blots with anti-O-GlcNAc antibodies to evaluate liver protein O-linked glycosylation profiles. (**C**) Enlarged 2D Western blot areas to highlight the effects of uridine on O-linked glycosylation profiles of protein spots 9, 10, and 30.

The effects of uridine salvage into UTP on liver glycogen and protein glycosylation were evaluated in C57BL/6J mice. Consistent with previous findings in skeletal muscles [11], dietary uridine supplementation at a daily dosage of 400 mg/kg for 5 days increased liver glycogen content by more than 2 folds (**Figure 1A**). To evaluate liver protein glycosylation profiles, total liver extracts were used for 2D Western blots, where proteins were separated by both charges and molecular weights (**Figure 1B**). Anti-O-GlcNAc monoclonal antibody was used to detect glycosylated liver proteins. Selective protein spots were excised and identified with matrix-assisted laser desorption/ionization time-of-flight mass spectrometry (MALDI-TOF-MS) (**Tables S1 & S2**). 2D Western blots revealed that uridine supplementation increased O-linked glycosylation of 10 protein spots. Of particular interest are the changes to several O-linked glycosylated protein spots with molecular weight of 60 kD (**Figure 1C** **, spots 9, 10, & 30**). Interestingly, MALDI-TOF-MS analysis identified the presence of an ER protein disulfide isomerase A3 (PDI) following uridine administration in protein spots 9, 10, and 30. In the liver of control mice, only O-linked glycosylated protein spots 9 and 10 were presence. However, three O-linked glycosylated protein spots 9, 10, and 30 were observed in the liver of mice treated with uridine. The presence of PDI at three locations with different isoelectric points suggests that PDI might have been post-translationally modified. Indeed, post-translational modification of PDI with N-linked glycosylation has been reported [20]. It should be noted that the presence of PDI in three O-linked glycosylated protein spots does not necessarily mean that PDI is O-linked glycosylated. Specific site of O-linked glycosylation of PDI has not been identified in the literature or in this study. Nonetheless, uridine treatment appeared to increase both liver glycogen biosynthesis and protein O-linked glycosylation in C57BL/6J mice.

**Figure 2:**
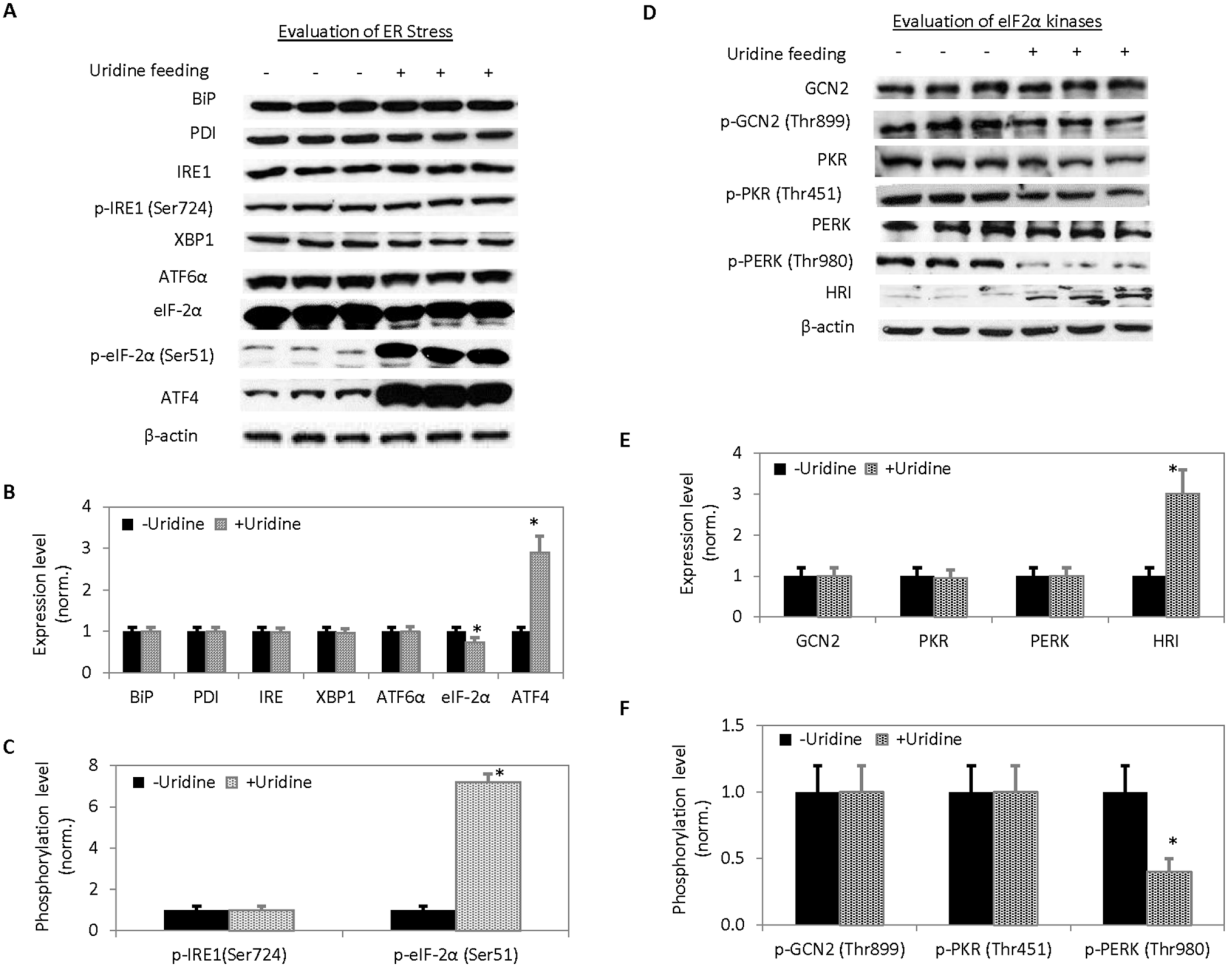
Evaluation of the effects of uridine on ER stress and regulation of eIF-2α. (**A**) Western blots of protein unfolded response sensors. (**B, C**) Quantitative analysis of protein expression levels (**B**) and phosphorylation levels (**C**) of UPR sensors. (**D**) Western blots of eIF-2α kinases. (**E, F**) Quantitative analysis of protein expression (**E**) and phosphorylation (**F**) levels of eIF-2α kinases. Error bars are standard deviations across 9 liver samples evaluated. Asterisks indicate P<0.05 versus untreated control C57BL/6J mice.

ER is a major site of protein folding and protein trafficking including glycoproteins [21]. Activation of the unfolded protein response (UPR) due to ER stress is linked to metabolic dysfunction including fatty liver disease [22], [23]. To evaluate the effects of uridine on ER function, expression and phosphorylation of UPR sensors were measured with Western blots. Among the proteins evaluated were binding immunoglobulin protein (BiP), which is also known as glucose-regulated protein 78 (GRP 78), PDI A3, inositol requiring 1α (IRE1), X-box binding protein 1 (XBP1), activating transcription factor 6α (ATF6α), eukaryotic translation initiation factor 2α (eIF-2α), and activating transcription factor 4 (ATF4). The liver expression levels of BiP, PDI A3, IRE, XBP1, and ATF6 were unchanged following uridine treatment (**Figure 2A, B**). The liver expression level of eIF-2α was reduced by approximately 30% following uridine treatment. In contrast, the liver expression level of ATF4 increased by nearly three folds. On the other hand, phosphorylation level of IRE1 protein at serine residue 724 was unchanged following uridine treatment (**Figure 2A, C**). In contrast, the phosphorylation level of eIF-2α protein at serine residue 51 increased by more than seven folds following uridine treatment. Uridine treatment did not have any effect on the liver protein expression and phosphorylation level of ER-localized UPR sensors. However, uridine treatment dramatically increased protein expression level of cytoplasm- and nucleus-localized ATF4 and phosphorylation level of cytoplasmic eIF-2α. Phosphorylation of eIF-2α at serine residue 51 inhibits protein synthesis at the translation initiation level [24]. A family of eIF-2α kinases is activated in response to various stress conditions including eIF-2α kinase 4 (GCN2 or EIF2AK4), protein kinase R (PKR or EIF2AK2), PKR-like ER kinase (PERK or EIF2AK3), and heme-regulated eIF-2α kinase (HRI or EIF2AK1) [25]. GCN2 is activated in response to amino acid starvation [26]. PKR is activated in response to viral infection [27]. PERK is activated in response to ER stress [28]. HRI is activated in response to heme deficiency [29]. To determine the stress condition and the eIF-2α kinases responsible for phosphorylating eIF-2α following uridine treatment, Western blots were performed on the expression or phosphorylation levels of all four eIF-2α kinases (**Figure 2D**). Following uridine treatment, the liver protein expression levels of three eIF-2α kinases GCN2, PKR, and PERK were unchanged while the expression level of HRI increased by approximately 3 folds (**Figure 2E**). HRI has a predicted molecular weight of approximately 71 kD. However, post-translational modification of HRI such as phosphorylation commonly results in a shift toward higher molecular weights [30]. Thus, HRI can appear in more than one gel band on a Western blot. Phosphorylation levels at selective threonine residues of liver GCN2 and PKR proteins were unchanged while phosphorylation level of PERK protein at threonine residue 980 was reduced by more than two folds due to uridine treatment (**Figure 2F**). Phosphorylation level of HRI was not evaluated because antibody against phosphorylated form of HRI was not commercially available. Western blot data indicated that increased phosphorylation of eIF-2α was likely a consequence of increased HRI expression.

**Figure 3:**
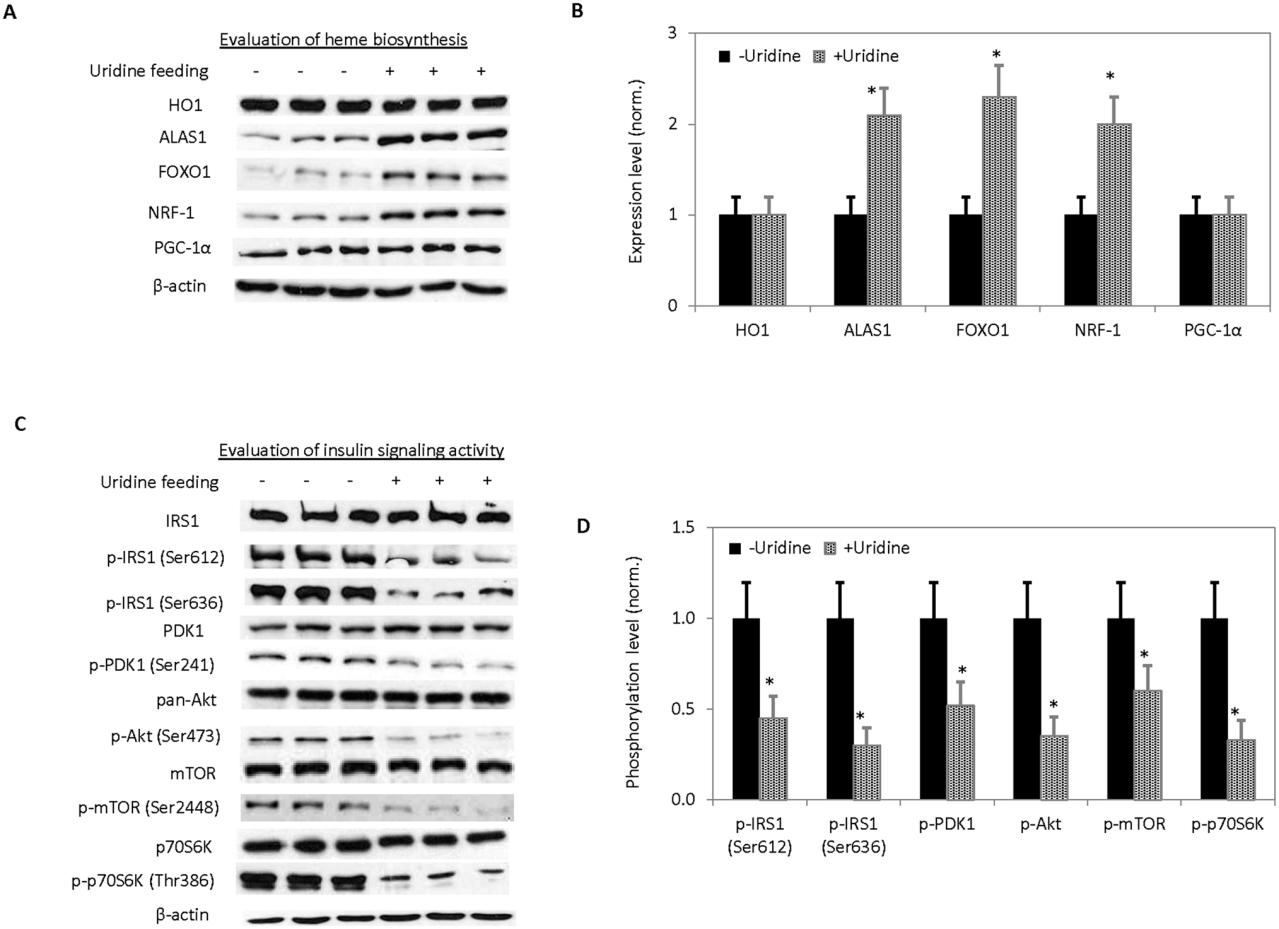
Evaluation of the effects of uridine on liver heme biosynthesis and insulin signaling activity. (**A**) Western blots of proteins participating in the regulation of heme biosynthesis and catabolism. (**B**) Quantitative analysis of protein expression levels of Western blot data presented in (**A**). (**C**) Western blots of proteins participating in the insulin signaling pathway. (**D**) Quantitative analysis of protein expression and phosphorylation levels of Western blot data presented in (**C**). Error bars are standard deviations across 9 liver samples evaluated. Asterisks indicate P<0.05 versus untreated control C57BL/6J mice.

To determine the cause of uridine-induced increases in HRI protein expression level, heme biosynthesis activity of the liver was examined through the expression levels of participating proteins including heme oxygenase 1 (HO1) and delta-aminolevulinate synthase 1 (ALAS1). HO1 is an enzyme that catabolizes free heme and ALAS1 is an enzyme that controls the rate-limiting step in hepatic heme biosynthesis [31], [32]. The promoter of a gene encoding for ALAS1 is regulated by peroxisome proliferator-activated receptor γ co-activator 1α (PGC-1α), forkhead box protein O1 (FOXO1), and nuclear respiratory factor 1 (NRF-1) [33], [34]. Using Western blots, the liver expression levels of HO1 and PGC-1α were unchanged following uridine treatment (**Figure 3A, B**). In contrast, the expression levels of ALAS1, FOXO1, and NRF-1 increased by over two folds following uridine treatment. Clearly, uridine treatment caused induction of hepatic ALAS1, thus, affecting liver heme biosynthesis.

To evaluate the relationship between uridine treatment and insulin resistance, the expression and phosphorylation levels of selective proteins in the insulin signaling pathway were measured with Western blots (**Figure 3C**). Following uridine treatment, there was no change to the liver protein expression levels of insulin receptor substrate (IRS), phosphoinositide-dependent protein kinase 1 (PDK1), Akt, mammalian target of rapamycin (mTOR), or p70S6 kinase (p70S6K). Interestingly, the phosphorylation levels of these proteins were reduced by at least 50% (**Figure 3D**). Thus, a consequence of uridine treatment was an overall reduction in the phosphorylation level of the liver insulin signaling proteins.

**Figure 4:**
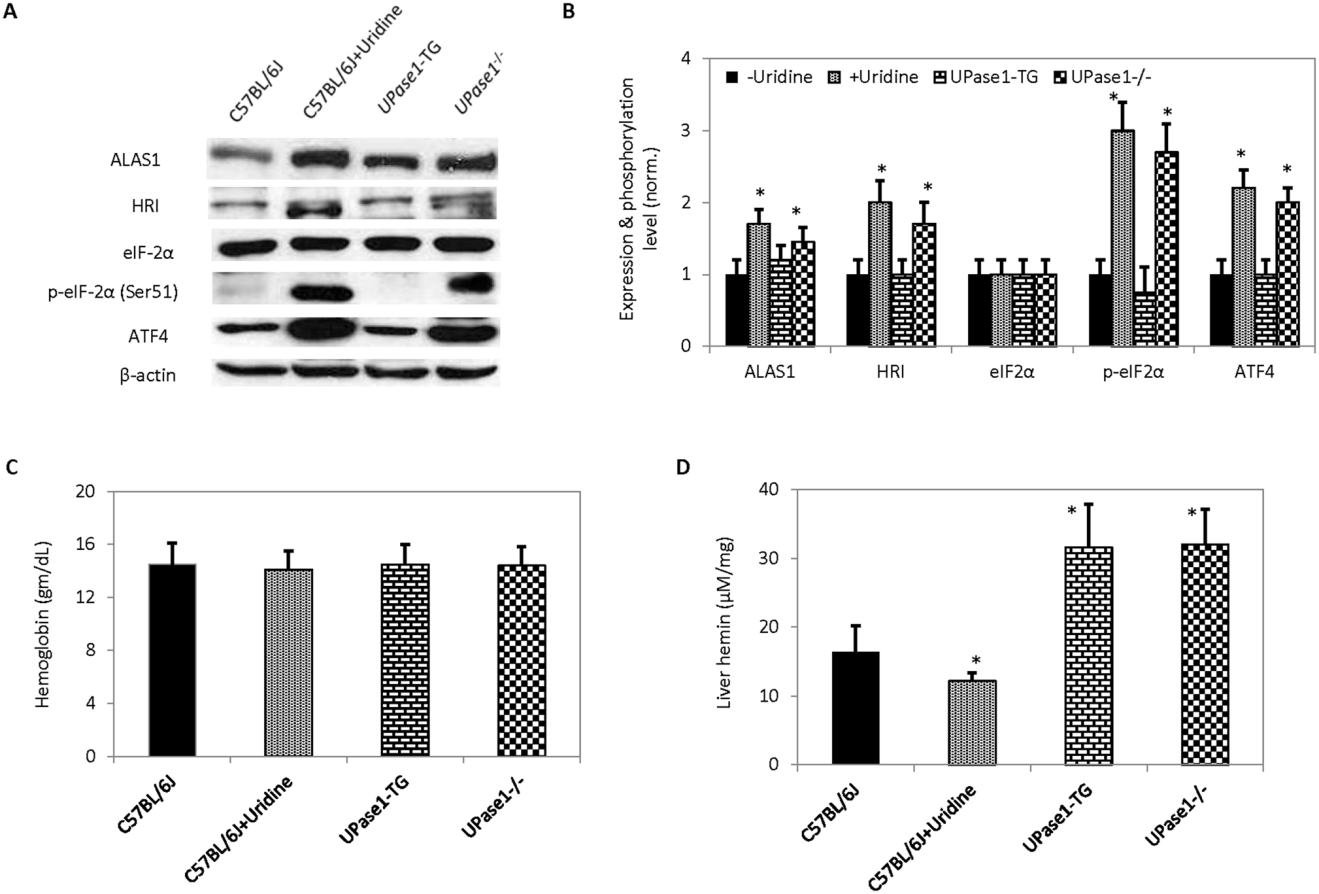
Evaluation of the effects of perturbations to uridine homeostasis on heme-deficiency response pathway. (**A**) Western blot analyses of protein participating in the heme-deficiency response pathway. (**B**) Quantitative analysis of Western blot data presented in (**A**). (**C**) Blood hemoglobin levels. (**D**) Liver hemin concentration. Error bars are standard deviations across blood or liver samples collected from 9 mice. Asterisks indicate P<0.05 versus untreated control C57BL/6J mice.

Next, transgenic *UPase1* ^−/−^ and *UPase1*-TG mice were employed to evaluate the chronic effects of uridine on liver heme-deficiency stress response. Consistent with the short-term effects of dietary uridine supplementation in C57BL/6J mice, *UPase1* ^−/−^ mice with long-term elevated levels of endogenous uridine concentration also exhibited increased liver expression levels of ALAS1, HRI, and ATF4, and increased phosphorylation level of eIF-2α at serine residue 51 (**Figure 4A, B**). In contrast, *UPase1*-TG mice with long-term depletion of endogenous uridine concentration exhibited comparable expression levels of liver ALAS1, HRI, ATF4 and phosphorylation levels of eIF-2α at serine residue 51 to control untreated C57BL/6J mice. Both short-term and long-term effects of elevated uridine levels on liver heme-deficiency stress response were conserved.

In addition, the effects of uridine on heme biosynthesis were evaluated in C57BL/6J and transgenic mice. There was no observable difference in blood hemoglobin levels between C57BL/6J mice without and with uridine treatment and transgenic mice (**Figure 4C**). However, dietary uridine treatment of C57BL/6J mice exhibited approximately 20% reduction in liver hemin level compared to untreated control C57BL/6J mice (**Figure 4D**). Interestingly, both *UPase1* ^−/−^ and *UPase1*-TG mice exhibited liver hemin levels at approximately two times higher than untreated control C57BL/6J mice. Liver hemin levels increased due to either chronic elevation or depletion of endogenous uridine concentration.

**Figure 5:**
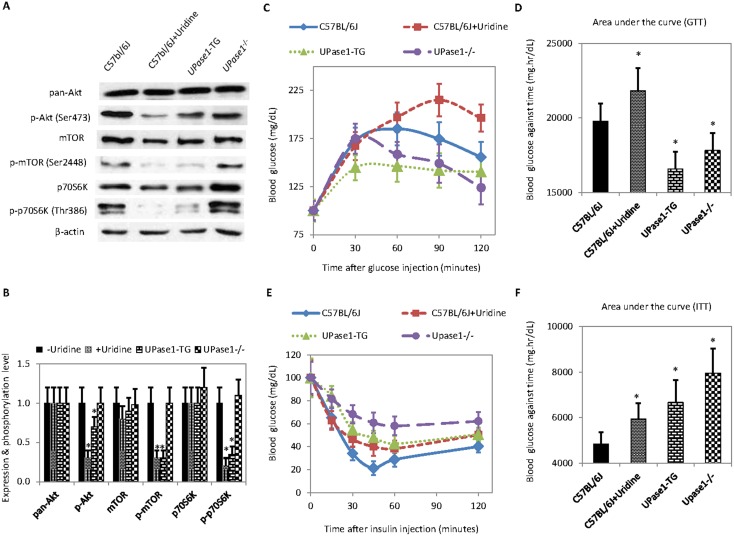
Evaluation of the effects of perturbations to uridine homeostasis on liver insulin signaling activity and blood glucose utilization. (**A**) Western blots of proteins participating in liver insulin signaling. (**B**) Quantitative analysis of protein expression and phosphorylation levels of Western blot data presented in (**A**). (**C**) Glucose tolerance test (GTT) to evaluate blood glucose levels as a function of time after glucose administration. (**D**) Integrated blood glucose level as a function of time after glucose administration. (**E**) Insulin tolerance test (ITT) to evaluate blood glucose level as a function of time after insulin administration. (**F**) Integrated blood glucose level as a function of time after insulin administration. Error bars are standard deviations across liver samples of 9 mice or blood samples of 12 mice. Areas under the curve were calculated using the trapezoidal rule. Asterisks indicate P<0.05 versus untreated control C57BL/6J mice.

Interestingly, the short-term and long-term effects of uridine on the phosphorylation level of liver insulin signaling proteins were not conserved. The phosphorylation levels of liver proteins Akt, mTOR, and p70S6K in *UPase1* ^−/−^ mice were comparable to control untreated C57BL/6J mice (**Figure 5A, B**). In contrast, *UPase1*-TG mice exhibited reduced phosphorylation levels of liver proteins Akt, mTOR, and p70S6K compared to control untreated C57BL/6J mice. The phosphorylation levels of liver proteins Akt, mTOR, and p70S6K in *UPase1*-TG mice resembled those of C57BL/6J mice treated with uridine. Contradicting effects of short-term and long-term uridine levels on the phosphorylation level of the liver insulin signaling proteins suggested possible adaptation to disruption of uridine homeostasis in transgenic *UPase1* ^−/−^ and *UPase1*-TG mice.

The effects of uridine on blood glucose utilization were also evaluated with glucose tolerance tests (GTT) in C57BL/6J and transgenic mice. In untreated control C57BL/6J mice, blood glucose level peaked at 60 minutes after intraperitonial injection of glucose (**Figure 5C**). In C57BL/6J mice treated with uridine, blood glucose level peaked at 90 minutes after glucose administration. Compared to untreated control C57BL/6J mice, C57BL/6J mice treated with uridine exhibited 15% higher average blood glucose levels at 120 minutes after glucose administration. Surprisingly, in *UPase1* ^−/−^ mice, blood glucose level peaked at 30 minutes after glucose administration. At 120 minutes after glucose administration, average blood glucose level of *UPase1* ^−/−^ mice was approximately 15% lower than that of control untreated C57BL/6J mice. On the other hand, in *UPase1*-TG mice, blood glucose level peaked at 30 minutes after glucose administration, but remained at peak level until 120 minutes. Integrated blood glucose as a function of time revealed that short-term treatment of C57BL/6J mice with uridine led to elevated blood glucose level following GTT compared to control C57BL/6J mice (**Figure 5D**). In contrast, long-term perturbation to uridine homeostasis in both *UPase1*-TG and *UPase1* ^−/−^ mice led to lowered blood glucose level following GTT compared to control C57BL/6J mice.

Insulin tolerance tests (ITT) were also conducted where blood glucose levels following insulin administration were monitored (**Figure 5E**). Following insulin administration, the blood glucose levels were substantially higher in C57BL/6J mice with short-term uridine treatment compared to control untreated C57BL/6J mice (**Figure 5E, F**). Interestingly, blood glucose levels in both *UPase1*-TG and *UPase1* ^−/−^ mice were substantially higher compared to control untreated C57BL/6J mice following insulin administration (**Figure 5E, F**). It appeared that both short-term and long-term perturbations to uridine homeostasis led to insensitivity to insulin-stimulated blood glucose removal in mice.

In addition, the effects of uridine administration on the expression and phosphorylation level of proteins participating in the metabolism of liver lipid, glycogen, glucose and glutamine were also examined (**Figure S1**). However, no significant effect was observed following the administration of uridine. The increase in liver glycogen content following uridine administration was likely a consequence of increased UDPG, which is a substrate for glycogen biosynthesis [10].

## Discussion

In this study, we report that uridine administration in C57BL/6J mice increases liver protein glycosylation profiles, reduces phosphorylation level of liver insulin signaling proteins, and activates the HRI-eIF-2α-ATF4 heme-deficiency stress response pathway. Uridine administration is associated with reduced ability to remove blood glucose during a glucose tolerance test and insensitivity to insulin-stimulated blood glucose removal during an insulin tolerance test. Uridine administration is also associated with a reduced liver hemin level while having no effect on the blood hemoglobin level.

In recent years, cross-talk between O-linked glycosylation and phosphorylation has been proposed as the basis for hyperglycemia-induced insulin resistance [35]. The serine and threonine residues of a protein are susceptible to post-translational modifications including phosphorylation and O-linked glycosylation [36]. The activities of important regulatory proteins such as Akt and FoxO1 have been shown to be regulated by both phosphorylation and O-linked glycosylation [37], [38]. It is important to point out that the activity and cellular distribution of FoxO1 is regulated by Akt [39]. FoxO1 is a transcriptional factor that controls the expression of ALAS1 [33], [40]. ALAS1 controls the rate-limiting step in heme biosynthesis. Overexpression of ALAS1 could cause accumulation of intermediates that activate heme-deficiency stress response via the HRI-eIF-2α-ATF4 signaling pathway [32]. Increased O-linked glycosylation of insulin signaling proteins has been shown to impair their activation in pancreatic β-cells [41]. In addition, FoxO1 has been shown to play a dual role in controlling hepatic insulin sensitivity and lipid metabolism [42]. It is plausible that uridine plays an indirect role in the cross-talk between O-linked glycosylation and phosphorylation of insulin signaling proteins and FoxO1 leading to the observed effects on liver metabolism. However, further studies are needed to delineate the precise links between uridine, liver protein O-linked glycosylation, insulin signaling activity, and heme biosynthesis.

Interestingly, some of the effects on liver metabolism by exogenous uridine supplementation on C57BL/6J mice are not conserved in transgenic *UPase1* ^−/−^ and *UPase1*-TG mice with disrupted endogenous uridine homeostasis. The non-conserved effects of uridine include the phosphorylation level of liver insulin signaling proteins and the liver hemin level. Given the importance of insulin signaling and heme production to the functions of the liver, it is conceivable that there are long-term adaptations to chronic perturbations to endogenous uridine homeostasis. A striking observation is the activation of the HRI-eIF-2α-ATF4 signaling pathway accompanying by an increase in liver hemin level in *UPase1* ^−/−^ mice compared to untreated control C57BL/6J mice. Increased liver hemin level is possible if the adaptation process in *UPase1* ^−/−^ and *UPase1*-TG mice involves either inhibition of liver hemin degradation or increased expression level of heme biosynthesis enzymes downstream of ALAS1. Transgenic *UPase1* ^−/−^ and *UPase1*-TG mice with disrupted endogenous uridine homeostasis provide suitable animal models for future studies of long-term effects of uridine on liver metabolism.

Purines and pyrimidines are complementary bases of DNA and RNA. Purines such as ATP and GTP and their derivatives are critical for signal transduction processes mediated by protein kinases [43]. Protein phosphorylation is a well-known mode of post-translational modification important for a wide range of cellular activities. ATP and GTP serve as key donor substrates for proteins and lipids phosphorylation. The roles of ATP and GTP in cellular functions have been well-investigated. In contrast, how pyrimidines CTP, UTP, and TTP contribute to cellular signal transduction and energy metabolism is currently unclear. We have reported a role of uridine in liver protein lysine acetylation in several recent studies [3], [7]. A previous study and this study highlight a role of uridine in liver protein O-linked glycosylation [11]. We report in this study the participation of uridine in liver insulin signaling pathway and heme biosynthesis. It is emerging that uridine and pyrimidine derivatives are contributing to cellular signal transduction and energy metabolism, perhaps in a complementary fashion to those of purines and their derivatives. Indeed, disruptions of pyrimidine nucleotide metabolism have been linked to multiple human malignancies [44]. Expression of the gene encoding for liver-specific uridine phosphorylase is highly regulated by lipid-sensing nuclear receptors [45]. The significance of pyrimidines and their derivatives in human physiology is clear, but their precise functions are not clearly understood. Future systematic and in-depth studies should be pursued to fill the gap in the current literature on the roles of pyrimidines in cellular functions.

## Materials and Methods

### Animals

All animal studies were performed in conformity with the Public Health Service Policy on Humane Care and Use of Laboratory Animals and with the approval of the Animal Care and Use Committees at Nevada Cancer Institute, Desert Research Institute, and Touro University Nevada. All mice used were male at 8–10 weeks of age. Mice were fed with PicoLab Mouse Diet ground pellets (Cat. No. 5058, LabDiet, Brentwood, MO) that provide 4.6 kcal/g and consist of 22% protein and 9% fat. The lipid composition includes cholesterol (200 ppm), linoleic acid (2.32%), linolenic acid (0.21%), arachidonic acid (0.02%), and omega-3 fatty acid (0.32%). The total saturated and monounsaturated fatty acids are 2.72% and 2.88%, respectively. Three mice strains were used for this study, C57BL/6J mice (Jackson Lab, Bar Harbor, Maine), and transgenic mice *UPase1* ^−/−^ and *UPase1*-TG with C57BL/6J background generated by our laboratory and described previously [3], [5]. Transgenic mice described in this study have been deposited into the Mutant Mouse Regional Resource Centers supported by the National Institutes of Health. The MMRRC strains are now known as B6; 129-*Upp1^tm1Gp^*/Mmucd (037119-UCD) for *UPase1* ^−/−^ mice and B6; FVB-*Gt(ROSA)26Sor^tm1.1(CAG-Upp1)Gp^*/Mmucd (037120-UCD) for *UPase1*-TG mice. For mice receiving uridine supplementation, uridine was thoroughly mixed with ground pellets with approximate daily dosage of 400 mg/kg. Mice were placed on control or supplemented diets for 5 days and were not fasted prior to terminal blood and liver samples collection in early mornings. Blood samples were collected via the tail veins while mice were under anesthesia with isoflurane. Cardiac perfusion under deep anesthesia with isoflurane was performed for liver tissue collection. Mice were anaesthetized and incisions were made from the abdomen up to the torso. Diaphragms were severed and 22 gauge needles were inserted into the left ventricles. Phosphate buffered saline (PBS) was used as the perfusate. Approximately 50–100 ml of PBS was flushed through each mouse from the left ventricle and exited through the incision made to the right atrium. Liver tissues were collected following the perfusion procedure.

### 1D Western Blots

Total liver protein extracts were separated on 10% SDS-PAGE gels, transferred to nitrocellulose membranes, incubated first with primary antibodies against proteins of interest and then with secondary antibodies conjugated with horseradish peroxidase. Membranes were developed with enhanced chemiluminescence reagents (Cat. No. 34075, Thermo Scientific), stripped, and re-incubated with antibodies against β-actin for evaluation of loading controls. Primary antibodies used are listed in Table S3. Representative Western blot data of 3 mice per strain per experimental condition were presented.

### Quantitative Analysis of 1D Western Blots

The liver samples of 9 mice per strain per experimental condition were used for 1D Western blot analysis. Quantitative analyses of Western blot data were performed using the NIH ImageJ software. Protein expression levels were adjusted with β-actin levels for loading controls and normalized to 1 for untreated control liver samples. Protein phosphorylation levels were adjusted with both protein expression levels and β-actin levels for loading controls and normalized to 1 for untreated control liver samples. Liver protein expression or phosphorylation levels for experimental animal groups were normalized correspondingly to those of untreated control animal group.

### 2D Western Blots

2D Western blots were performed by Kendrick Laboratories (Madison, WI). Approximately 500 µg of protein from each liver tissue was loaded per gel. Primary anti-acetylated lysine antibodies were provided by Kendrick Lab. Proteins were separated using isoelectric focusing (IEF) in the first dimension and SDS polyacrylamide gel electrophoresis (SDS-PAGE) in the second dimension. Isoelectric focusing was carried out in a glass tube of inner diameter 3.3 mm using 2.0% pH 4–8 mix Servalytes (Serva, Heidelberg, Germany; and 2 mM lysine) for 20,000 volt-hrs. After equilibration for 10 min in 10% glycerol, 50 mM dithiothreitol, 2.3% SDS and 0.0625 M tris, pH 6.8, each tube gel was sealed to the top of a stacking gel that overlaid a 10% acrylamide slab gel (1.00 mm thick). SDS slab gel electrophoresis was carried out for about 5 hrs at 25 mA/gel. The following proteins (Sigma Chemical Co., St. Louis, MO) were used as molecular weight standards: myosin (220 kD), phosphorylase A (94 kD), catalase (60 kD), actin (43 kD), carbonic anhydrase (29 kD) and lysozyme (14 kD). The liver samples of 3 random C57BL/6J mice or C57BL/6J mice treated with uridine were used for 2D Western blots for triplicate analysis.

### Protein Identification with MALDI-TOF-MS

Immuno-positive protein spots were identified and corresponding protein spots from duplicate gels were picked and sent to Applied Biomics (Hayward, CA) for identification with matrix-assisted laser desorption/ionization time-of-flight mass spectrometry (MALDI-TOF-MS).

### Liver Glycogen Measurement

Liver glycogen content was measured using an enzymatic assay kit according to manufacturer’s protocols (Cat. No. 700480, Cayman Chemical, Ann Arbor, MI) and normalized with liver weight.

### Liver Hemin Measurement

Liver hemin concentration was measured using an enzymatic assay kit according to manufacturer’s protocols (Cat. No. ab65332, Abcam, Cambridge, MA) and normalized with liver weight.

### Hemoglobin Measurement

Blood was drawn from the tail vein and hemoglobin level was determined using a hemoglobin meter (Cat. No. 900900, Stanbio Laboratory, Boerne, TX).

### Glucose Tolerance Test

Mice were fasted for 5 hours, then given an intraperitoneal injection of 10% d-glucose at 0.75 g/kg. Blood was drawn from the tail vein at 0, 30, 60, 90, 120 minutes and assayed for glucose level using a glucose meter (Cat. No. 7151G, Bayer, Leverkusen, Germany). Due to variation in the initial blood glucose levels among fasted mice, the blood glucose levels at 0 minute were normalized to 100 mg/dL and correspondingly for other timepoints for all mice groups.

### Insulin Tolerance Test

Mice were fasted for 5 hours, then given an intraperitoneal injection of insulin at 0.5 U/kg. Blood was drawn from the tail vein at 0, 15, 30, 45, 60, and 120 minutes and assayed for glucose level using a glucose meter. Due to variation in the initial blood glucose levels among fasted mice, the blood glucose levels at 0 minute were normalized to 100 mg/dL and correspondingly for other timepoints for all mice groups.

### Statistical Analysis

Data were presented as average values ± standard deviations. Statistical analysis was performed using Excel’s paired Student’s t-test and analysis of variance (ANOVA) functions for experimental versus control animal groups. Statistical significance was set at p≤0.05 versus control untreated C57BL/6J mice.

## Supporting Information

Figure S1 **Evaluation of the effects of uridine on the metabolism of lipid, glycogen, glucose, and glutamine.** (**A**) Western blots of proteins participating in liver lipid and glycogen metabolism. (**B**) Quantitative analysis of protein expression and phosphorylation levels of Western blot data presented in (**A**). (**C**) Western blots of proteins participating in liver glucose and glutamine metabolism. (**D**) Quantitative analysis of protein expression and phosphorylation levels of Western blot data presented in (**C**). Error bars are standard deviations across 9 liver samples evaluated. Asterisks indicate P<0.05 versus untreated control C57BL/6J mice.
(TIF)

Table S1 **Protein identification with MALDI-TOF-MS.** *The proteins presented in Table S1 were present in the excised gel spots which were found to be immune-positive with anti-O-linked N-acetylglucosamine antibodies. Their O-linked glycosylation sites have not been identified with MALDI-TOF-MS. Proteins that migrated at molecular weights different from predicted molecular weights were likely due to post-translational modifications or degradation.
(DOCX)

Table S2 **MALDI-TOF-MS data summary.**
(DOCX)

Table S3 **List of primary antibodies.**
(DOCX)
