# Supplementary figures and images for: Uridine Affects Liver Protein Glycosylation, Insulin Signaling, and Heme Biosynthesis
Source: PLoS One. 2014 Jun 11;9(6):e99728. doi: 10.1371/journal.pone.0099728 (PMC4053524; doi:10.1371/journal.pone.0099728)

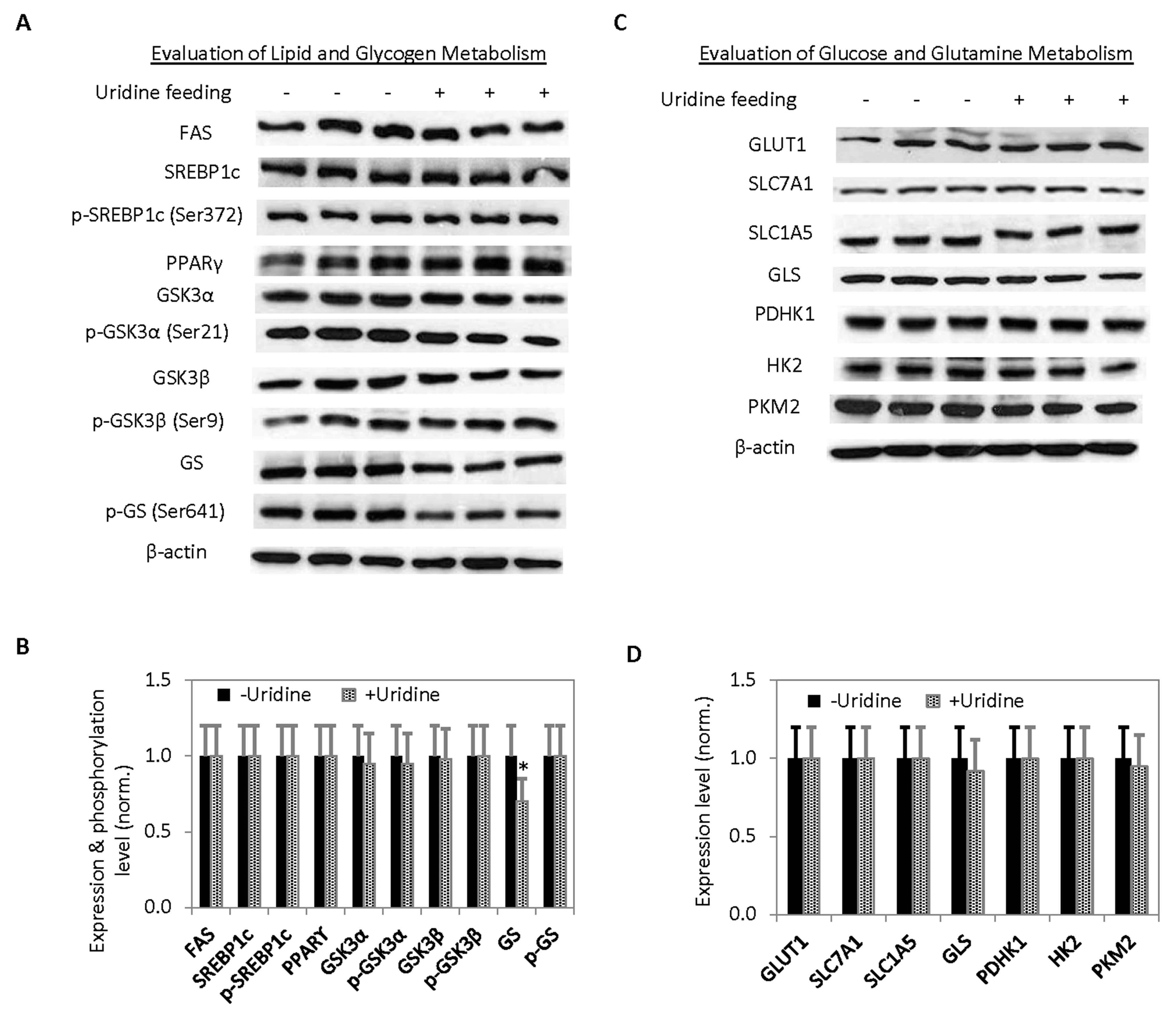

Supplement: Figure S1 — Evaluation of the effects of uridine on the metabolism of lipid, glycogen, glucose, and glutamine. (A) Western blots of proteins participating in liver lipid and glycogen metabolism. (B) Quantitative analysis of protein expression and phosphorylation levels of Western blot data presented in (A). (C) Western blots of proteins participating in liver glucose and glutamine metabolism. (D) Quantitative analysis of protein expression and phosphorylation levels of Western blot data presented in (C). Error bars are standard deviations across 9 liver samples evaluated. Asterisks indicate P<0.05 versus untreated control C57BL/6J mice. (TIF) [file pone.0099728.s001.tif]
