# Supplementary material for: Uridine Affects Liver Protein Glycosylation, Insulin Signaling, and Heme Biosynthesis
Source: PLoS One. 2014 Jun 11;9(6):e99728. doi: 10.1371/journal.pone.0099728 (PMC4053524; doi:10.1371/journal.pone.0099728)
Supplement: Table S1 — Protein identification with MALDI-TOF-MS. *The proteins presented in Table S1 were present in the excised gel spots which were found to be immune-positive with anti-O-linked N-acetylglucosamine antibodies. Their O-linked glycosylation sites have not been identified with MALDI-TOF-MS. Proteins that migrated at molecular weights different from predicted molecular weights were likely due to post-translational modifications or degradation. (DOCX) [file pone.0099728.s002.docx]

**Table S1.** **Proteins identification with MALDI-TOF-MS**

| **Spot #** | **Protein Name** | **C57BL/6J** | **C57BL/6J+U** | **Biological Process** |
| --- | --- | --- | --- | --- |
| **1** | Regucalcin | √ | √ | Calcium Homeostasis |
| **2** | Regucalcin | √ | √ | Calcium Homeostasis |
| **3** | Indolethylamine N-methyltransferase | √ | √ | Detoxification |
| **4** | 3-hydroxyanthranilate 3,4-dioxygenase | √ | √ | NAD Biosynthesis |
| **5** | Adenosylhomocysteinase | √ | √ | One-carbon Metabolism |
| **6** | 4-hydroxyphenylpyruvate dioxygenase | √ | √ | Phe/Tyr Catabolism |
| **7** | 4-trimethylaminobutyraldehyde dehydrogenase | √ | √ | Carnitine Biosynthesis |
| **8** | Aldehyde dehydrogenase, mitochondrial | √ | √ | Detoxification |
| **9** | Protein disulfide-isomerase A3 | √ | √ | Protein Folding |
| **10** | Protein disulfide-isomerase A3 | √ | √ | Protein Folding |
| **11** | Replication factor C subunit 5 | √ | √ | DNA Replication |
| **12** | 60 kDa heat shock protein, mitochondrial | √ | √ | Stress Response |
| **13** | 60 kDa heat shock protein, mitochondrial | √ | √ | Stress Response |
| **14** | Succinate dehydrogenase [ubiquinone], mitochondrial | √ | √ | Electron Transport |
| **15** | Succinate dehydrogenase [ubiquinone], mitochondrial | √ | √ | Electron Transport |
| **16** | Dimethylglycine dehydrogenase, mitochondrial | √ | √ | Glycine Catabolism |
| **17** | Elongation factor 2 | √ | √ | Protein Synthesis |
| **18** | Sarcosine dehydrogenase, mitochondrial | √ | √ | Glycine Catabolism |
| **19** | Sarcosine dehydrogenase, mitochondrial | √ | √ | Glycine Catabolism |
| **20** | Cytosolic 10-formyltetrahydrofolate dehydrogenase | √ | √ | One-carbon Metabolism |
| **21** | 3-oxo-5-beta-steroid 4-dehydrogenase | - | √ | Steroid Metabolism |
| **22** | Glycine N-methyltransferase | - | √ | One-carbon Metabolism |
| **23** | Ornithine carbamoyltransferase, mitochondrial | - | √ | Amino Acid Biosynthesis |
| **24** | Short-chain specific acyl-CoA dehydrogenase, mitochondrial | - | √ | Lipid Metabolism |
| **25** | Fumarylacetoacetase | - | √ | Phe/Tyr Catabolism |
| **26** | S-formylglutathione hydrolase | - | √ | Formaldehyde Catabolism |
| **27** | Electron transfer flavoprotein subunit alpha, mitochondrial | - | √ | Electron Transport |
| **28** | Pyrroline-5-carboxylate reductase 3 | - | √ | Amino Acid Biosynthesis |
| **29** | Keratin, type II cytoskeletal 74 | - | √ | Cytoskeleton |
| **30** | Protein disulfide-isomerase A3 | - | √ | Protein Folding |
