# Supplementary material for: Uridine Affects Liver Protein Glycosylation, Insulin Signaling, and Heme Biosynthesis
Source: PLoS One. 2014 Jun 11;9(6):e99728. doi: 10.1371/journal.pone.0099728 (PMC4053524; doi:10.1371/journal.pone.0099728)
Supplement: Table S2 — MALDI-TOF-MS data summary. (DOCX) [file pone.0099728.s003.docx]

**Table S2.** **MALDI-TOF-MS data summary**
